# Preferred patient-rated outcome measure in patients with femoroacetabular impingement: a comparison between selected instruments

**DOI:** 10.1093/jhps/hnv057

**Published:** 2015-08-12

**Authors:** Franco M. Impellizzeri, Florian D. Naal, Anne F. Mannion, Michael Leunig

**Affiliations:** 1. Department of Research and Development, Schulthess Clinic, Lengghalde 2, 8008 Zurich, Switzerland; 2. Department of Orthopaedic Surgery, Schulthess Clinic, 8008 Zurich, Switzerland

## Abstract

The first aim of this study was to establish which questionnaire patients with femoroacetabular impingement (FAI) most often preferred out of a set of self-reported generic and region/joint-specific outcome measures. A second aim was to evaluate their preferred type of response scale. One hundred and sixty-two consecutive FAI patients undergoing surgery (51% females, age 32 [SD 12] years, body mass index 24 [SD 4] kg/m^2^) completed five specific questionnaires [Hip Outcome Score (HOS), Oxford Hip Score (OHS), Hip disability and Osteoarthritis Outcome Score, self-administered Harris Hip Score and Western Ontario and McMaster Universities Arthritis Index] and three generic questionnaires (WHO Quality of Life-BREF, EuroQoL and 12-Item Short Form Survey). In addition, the patients completed the International Physical Activity Questionnaire, a questionnaire on expectation, and two sports activity scales (TEGNER and UCLA). Patients were asked to indicate the questionnaires that best reflected their situation, the most difficult to complete, and had the preferred response scale. 64% indicated a joint specific questionnaire as the one that best addressed their situation, with 27 and 20% choosing the HOS and the OHS, respectively. Most patients (62%) expressed no difficulties completing the questionnaires: just 12% considered the IPAQ difficult to complete, and 6% the HOS. The preferred response scale was the adjectival scale (57%), compared with the Numeric Rating Scale (39%) and the VAS (4%). This study showed that patients with FAI consider joint-specific instruments to be most relevant to them, in particular the HOS and OHS, and generally prefer responding on an adjectival scale.

## INTRODUCTION

The use of self-report measures in orthopaedic patients is recommended for the assessment of pain and function in research and clinical routine. However, there are numerous instruments available for different diagnoses, joints and conditions. To the best of our knowledge, only the Hip Outcome Score (HOS) has been specifically developed and validated in patients suffering from femoroacetabular impingement (FAI) [1], although various questionnaires developed for other hip disorders (e.g. osteoarthritis) have already been used and are considered valuable for use in these patients [2].

It has been suggested that, when developing any new outcome tool, the involvement of the patients during item generation guarantees content validity of the questionnaire [3]. However, patients were not involved in the item generation of any of the instruments used in previous outcome studies in FAI. To better understand whether some instruments used in the literature really address the complaints of FAI patients, this study focused on patients’ preferences regarding questionnaire content and response options. Although this approach cannot overcome the lack of patient involvement in item generation, it can give useful insights regarding the content validity of existing hip questionnaires reported in the literature.

The aims of this study were to identify (1) the questionnaire(s) considered by FAI patients to be most relevant to them out of a large set of instruments (generic and joint/condition specific) and (2) the preferred response option(s) [adjectival scale, numerical scale or visual analogue scale (VAS)].

## METHODS

### Participants and study design

For this study we included 162 consecutive FAI patients undergoing surgery (56% hip arthroscopy, 44% mini-open approach or surgical hip dislocation; all ASA I and II; 49% males, 51% females; mean age 32.3 [SD 11.7] years; body mass index 23.7 [3.7] kg/m^2^). All demonstrated either cam, localized pincer or mild-to-moderate mixed hip impingement with at most early osteoarthritis (<1). They commonly presented with moderate to severe groin pain, usually exacerbated by physical activity and prolonged sitting, not resolved by conservative treatment. Symptoms had been present for about 6 months to 2 years. The diagnosis of impingement was based on patient history, clinical examination findings (reduced flexion and internal rotation and positive impingement test), conventional radiographs (anteroposterior pelvis and cross-table lateral views) and obligatory magnetic resonance imaging of the involved hip with intraarticular gadolinium contrast.

The patients completed several generic and specific questionnaires (see below). The order of the questionnaires was randomized. The study was approved by the local Ethics Committee and all participants gave their signed informed consent to participate.

### Questionnaires

The specific questionnaires used were: HOS [4], Oxford Hip Score (OHS) [5], Hip disability and Osteoarthritis Outcome Score (HOOS) [6], the self-administered Harris Hip Score (HHS) [7] and Western Ontario and McMaster Universities Arthritis Index (WOMAC) [8]. The HOOS comprises all WOMAC items and additional questions for assessing function in sport and recreation, and hip-related quality of life [6]; for this study, we generated a special form including only the extra items specific to the HOOS. Patients were also asked to complete three generic questionnaires: World Health Organization Quality of Life-BREF (WHOQOL-BREF [9], EuroQoL-5D [10] and 12-Item Short Form Survey (SF12)). In addition to these questionnaires the patients completed the International Physical Activity Questionnaire [11], a questionnaire on expectations [12] and two sports activity scales: Tegner scale and University of California at Los Angeles activity scale (UCLA) [2].

### Patient preferences

Patient preferences were assessed by a separate form consisting of three additional questions: (1) Which questionnaire best addresses your situation? (2) Which questionnaire was most difficult for you to answer? (3) Which response scale do you prefer (i.e. which is the easiest to understand and use)? An example of each scale was provided. The adjectival scale comprised five response options ranging from e.g. ‘no pain’ to ‘extreme pain’. The numeric scale ranged from 0 (e.g. no pain) to 10 (e.g. extreme pain). The VAS was 10 cm long starting from e.g. ‘no pain’ to ‘extreme pain’.

### Statistical analysis

Data are presented for descriptive purposes as percentage of respondents. For examining the difference between observed and expected proportions for each response option we used the *z*-test for one proportion were the expected proportion has been calculated based on the number of response options. Chi-square was also used to compare the proportions each other. The analyses were conducted using MedCalc (MedCalc Statistical Software, Mariakerke, Belgium). Probability values <0.05 were considered to be statistically significant.

## RESULTS

**Fig. 1.**
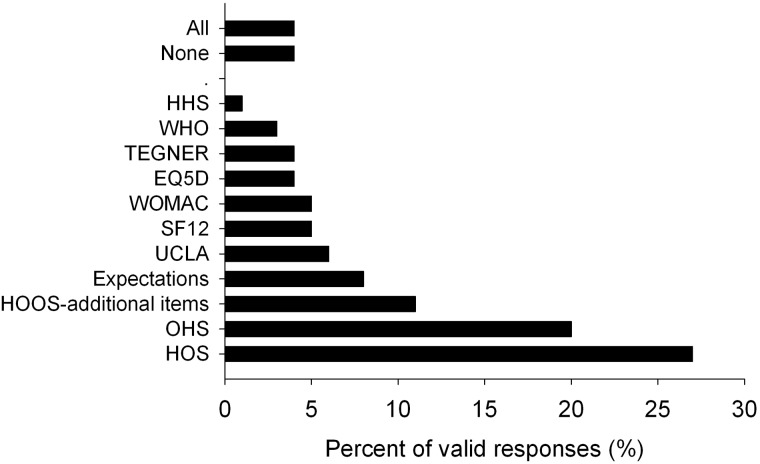
Frequency of responses regarding the questionnaire that best addressed the patient’s situation. HOS, Hip Outcome Score; OHS, Oxford Hip Score; HOOS, Hip disability and Osteoarthritis Outcome Score; HHS, self administered Harris Hip Score; WOMAC, Western Ontario and McMaster Universities Arthritis Index; WHO, World Health Organization Quality of Life-BREF; EQ5D, EuroQoL-5D; SF12, 12-Item Short Form Survey; IPAQ, International Physical Activity Questionnaire; Expectations, questionnaire asking for treatment expectations; UCLA, University of California Los Angeles Activity Scale; TEGNER, sport activity scale; All, all questionnaires.

Forty-five patients (28%) answered ‘I don’t know’ when asked which questionnaire best addressed their situation, and nine (6%) did not answer. Figure 1 shows the instruments considered most relevant by the remaining 108 patients. The HOS (27%), an instrument specifically designed for FAI patients, and the OHS (20%) designed for hip OA patients, were the questionnaires considered most relevant by FAI patients being the proportion of patients indicating the HOS and OHS the only ones significantly higher than the percentages expected by chance (*P* < 0.001 and *P* = 0.038, respectively). Overall a joint-specific instrument was considered most relevant by 64% patients when compared with the generic instruments (11%, *P* < 0.001), and a sports scale (9%, *P* < 0.001).

**Fig. 2.**
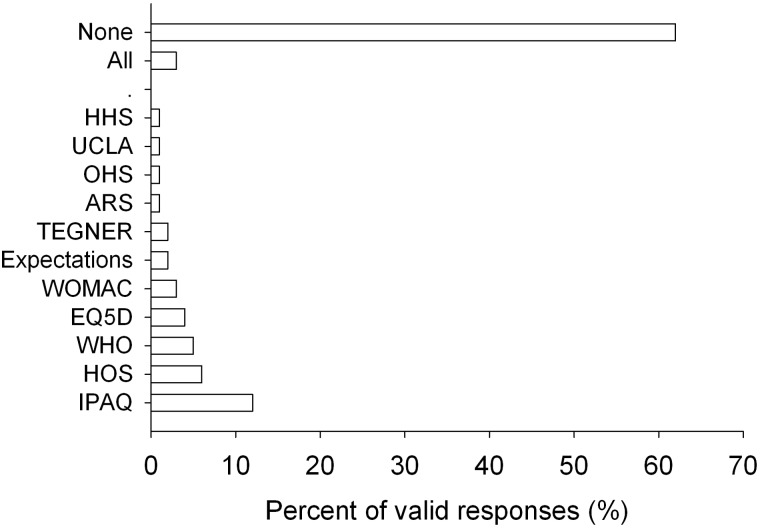
Frequency of responses regarding the questionnaire that was considered the most difficult to answer. HOS, Hip Outcome Score; OHS, Oxford Hip Score; HOOS, Hip disability and Osteoarthritis Outcome Score; HHS, self administered Harris Hip Score; WOMAC, Western Ontario and McMaster Universities Arthritis Index; WHO, World Health Organization Quality of Life-BREF; EQ5D, EuroQoL-5D; SF12, 12-Item Short Form Survey; IPAQ, International Physical Activity Questionnaire; Expectations, questionnaire asking for treatment expectations; UCLA, University of California Los Angeles Activity Scale; TEGNER, sport activity scale; All, all questionnaires.

Forty-six patients (28%) responded with ‘I don’t know’ when asked which was the most difficult to answer, and 12 (7%) did not answer. The majority (62%; *P* < 0.001) of the patients reported no difficulty filling any of the questionnaires (Fig. 2).

The preferred response scale was the adjectival scale (57%, *P* < 0.001), which was also the preferred compared with both the Numeric Scale (39%; *P* = 0.047) and the VAS (4%; *P* = 0.036).

## DISCUSSION

Among the various questionnaires used in this study, the HOS and the OHS were considered the instruments that best addressed the patients’ situation, i.e. the problems caused by FAI, enjoying almost equal popularity. All the other questionnaires, including the additional HOOS items and the WOMAC, were less frequently considered the most relevant. While for the HOS this result could be expected, the OHS was not specifically developed for FAI or for a younger population. Despite this, it would appear that the items of the OHS adequately address the major concerns of FAI patients, or at least address them to the same extent as the HOS does. The preference for the HOS and OHS indirectly supports the content validity of each. The study also suggests that examination of the relevance of a questionnaire as a whole rather than its single items may provide interesting and unexpected findings about its content validity and indirectly about the potential response rate. Indeed, it has been shown that questionnaires considered more interesting have a higher chance of being completed and returned (odds ratio: 2.44) (Edwards). The results of this study are interesting because the OHS is a short questionnaire and for this reason it is well suited to being included in national joint registries, having a reduced administrative burden [13]. However, unlike the HOS, the OHS does not include a sport domain, suggested to be a relevant domain for FAI patients. This weakness of the OHS may be partially compensated for by the use of an additional activity scale. A recent study has proposed a new activity scale (the Hip Sports Activity Scale) that has been purposely developed for FAI patients [14].

Among the specific questionnaires the HHS, although in the self-administered form (i.e. without the physical examination items), was rated as the least relevant, suggesting that this measure is not appropriate for evaluating FAI patients. Interestingly, this questionnaire has recently been suggested to be suboptimal even in the hip arthroplasty population for which it was originally developed [15].

The second research question enquired about the difficulty of completion of the questionnaires. This is another characteristic that may be useful for selecting the most suitable instrument (e.g. in terms of impact on missing data and response rates). Most of the patients reported no difficulties with any of the questionnaires, with a small proportion indicating difficulty completing the HOS, the IPAQ and some generic questionnaires. Of the response scales, the adjectival scale was the most popular, followed by the numeric scale. A very low percentage liked the VAS best. Therefore, the ideal questionnaire should employ adjectival scales or, alternatively, numeric scales, but not VAS for its response options.

Although we selected questionnaires already used in clinical studies on FAI patients, more recent instruments such as the Copenhagen Hip and Groin Outcome Score [2] and the International Hip Outcome Tool [16] were not included. The first is, however, based on the HOOS and the second is not available in German. Therefore, further studies are needed to assess patient preferences in relation to these newly developed instruments. Furthermore, although we enquired separately about relevance, difficulty and scale preference, we cannot exclude the possibility that, when indicating the questionnaire that best addressed their situation, patients were also partly influenced by the instrument length, response option and overall clarity. Finally, the findings of this study might be different in patients who are less well educated or informed and cannot be automatically extended to a population of different age.

In conclusion, this study showed that out of several questionnaires previously used in individuals with hip disorders, the specific questionnaires and particularly the HOS and OHS were considered by patients with FAI to be the instruments that best addressed their situation and were most relevant to them. The adjectival scale was the preferred scale for presenting the response options. Slightly more patients reported difficulties answering the HOS than the OHS. This suggests that the OHS might represent a potentially interesting instrument for use in the assessment of FAI patients. A recent study has shown that the measurement properties of the OHS in patients with FAI are comparable to those in patients with THA, supporting its potential validity also in this patient group [17]. This may obviate the need for different assessment tools for these populations of hip patients and would facilitate outcome assessment in large centres dealing with both pathologies.
